# Childhood vaccination coverage and equity impact in Ethiopia by socioeconomic, geographic, maternal, and child characteristics

**DOI:** 10.1016/j.vaccine.2020.03.040

**Published:** 2020-04-29

**Authors:** Anne Geweniger, Kaja M. Abbas

**Affiliations:** a Center for Paediatrics and Adolescent Medicine, University Medical Center, Faculty of Medicine, University of Freiburg, Germany; b Faculty of Public Health and Policy, London School of Hygiene & Tropical Medicine, United Kingdom; c Department of Infectious Disease Epidemiology, London School of Hygiene & Tropical Medicine, United Kingdom

**Keywords:** Immunisation coverage, Vaccine equity, Ethiopia, Demographic and health survey

## Abstract

**Background:**

Ethiopia is a priority country of Gavi, the Vaccine Alliance to improve vaccination coverage and equitable uptake. The Ethiopian National Expanded Programme on Immunisation (EPI) and the Global Vaccine Action Plan set coverage goals of 90% at national level and 80% at district level by 2020. This study analyses full vaccination coverage among children in Ethiopia and estimates the equity impact by socioeconomic, geographic, maternal and child characteristics based on the 2016 Ethiopia Demographic and Health Survey dataset.

**Methods:**

Full vaccination coverage (1-dose BCG, 3-dose DTP3-HepB-Hib, 3-dose polio, 1-dose measles (MCV1), 3-dose pneumococcal (PCV3), and 2-dose rotavirus vaccines) of 2,004 children aged 12–23 months was analysed. Mean coverage was disaggregated by socioeconomic (household wealth, religion, ethnicity), geographic (area of residence, region), maternal (maternal age at birth, maternal education, maternal marital status, sex of household head), and child (sex of child, birth order) characteristics. Concentration indices estimated wealth and education-related inequities, and multiple logistic regression assessed associations between full vaccination coverage and socioeconomic, geographic, maternal, and child characteristics.

**Results:**

Full vaccination coverage was 33.3% [29.4–37.2] in 2016. Single vaccination coverage ranged from 49.1% [45.1–53.1] for PCV3 to 69.2% [65.5–72.8] for BCG. Wealth and maternal education related inequities were pronounced with concentration indices of 0.30 and 0.23 respectively. Children in Addis Ababa and Dire Dawa were seven times more likely to have full vaccination compared to children living in the Afar region. Children in female-headed households were 49% less likely to have full vaccination.

**Conclusion:**

Vaccination coverage in Ethiopia has a pro-advantaged regressive distribution with respect to both household wealth and maternal education. Children from poorer households, rural regions of Afar and Somali, no maternal education, and female-headed households had lower full vaccination coverage. Targeted programmes to reach under-immunised children in these subpopulations will improve vaccination coverage and equity outcomes in Ethiopia.

## 1 Introduction

Ethiopia had a total population of 109 million with gross national income per capita of US$ 790 in 2018 [1]. Children under the age of 5 years constitute 14.9% of the total population, with an infant mortality rate of 41 per 1000 live births and under-five mortality rate of 59 per 1000 births [2], [3]. Vaccine preventable diseases are an important cause of childhood mortality with an under-five mortality of 25,970 deaths due to lower respiratory infections and 14,662 deaths due to diarrheal diseases in 2015 [4].

Vaccines are considered one of the safest and most cost-effective interventions to reduce childhood morbidity and mortality [5]. Globally, 2–3 million deaths are currently prevented by vaccination every year [6]. However, at present 19.4 million children under the age of one year do not receive basic vaccines, 60% of whom live in Angola, Brazil, Congo, Ethiopia, India, Indonesia, Nigeria, Pakistan, Philippines, and Vietnam [7].

### 1.1 Equity in vaccination coverage

Vaccination coverage is monitored as part of Sustainable Development Goal 3, “Ensure healthy lives and promote well-being for all at all ages”, which aims at ending preventable deaths of newborns and children under the age of 5 years by 2030, while the goal of SDG 10 is to “Reduce inequality within and among countries” [8]. Further, 14 of the 17 SDGs are related to the success of immunisation programmes in improving vaccination coverage and equitable uptake [9].

The Global Vaccine Action Plan 2011–2020 includes equitable access to immunisations among its six guiding principles and strategic objectives, advocating for the monitoring of equity indicators, such as the gap in vaccination coverage between the highest and lowest wealth quintiles [5]. Gavi, the Vaccine Alliance, has facilitated the improvement of immunisation access in the poorest countries through public–private partnerships, and incorporates measures of vaccine equity relating to geographic distribution, wealth, and maternal education in its 2016–2020 vaccine goal indicators [10].

The Ethiopian National Expanded Programme on Immunisation (EPI) aligns with the Sustainable Development Goals 2016–2030, the Global Vaccine Action Plan 2011–2020, and Gavi, the Vaccine Alliance in setting equity goals for health outcomes and vaccination coverage. The EPI and the Global Vaccine Action Plan have set coverage goals of 90% at national level and 80% at district levels by 2020.

### 1.2 Expanded programme on immunisation in Ethiopia

Ethiopia’s EPI was launched in 1980, and the routine immunisation schedule comprises six vaccine preventable diseases, namely measles, diphtheria, pertussis, tetanus, polio, and tuberculosis. Ethiopia has received support from Gavi since 2002 [11], and is one of ten tier-1 Gavi priority countries with the lowest national coverage for three doses of the combined diphtheria, tetanus toxoid, and pertussis vaccine (DTP3), a commonly used indicator for immunisation programme performance [12], [13]. Rotavirus, pneumococcal, and second dose of measles vaccines were introduced in 2011, 2013, and 2019 respectively [14], [15]. The comprehensive multi-year plan (cMYP 2016–2020) of Ethiopia’s National Expanded Programme on Immunisation states its first objective is to “reach 90% national coverage and 80% in every district with all vaccines by 2020” [16].

### 1.3 Study objective

Our aim is to analyse full vaccination coverage among children in Ethiopia and to estimate the equity impact by socioeconomic, geographic, maternal, and child characteristics based on the Ethiopia Demographic and Health Survey 2016 dataset. Aggregate country-level analysis conceals the hidden inequities in vaccination coverage, and the disaggregated equity impact analysis of this study will be valuable for identifying the underserved subpopulations and informing policies and practices to address the vaccination barriers specific to these subpopulations [17].

## 2 Methods

### 2.1 Survey data

The Demographic and Health Surveys (DHS) programme collects nationally representative data on population health in low and middle income countries [18]. The surveys provide estimates of key demographic and health indicators, covering population, maternal and child health issues with a special focus on marriage, sexual and reproductive health, child health, nutrition, and HIV/AIDS.

We used the Ethiopia Demographic and Health Survey (EDHS) 2016 dataset to analyse childhood vaccination coverage and to estimate equity outcomes [19]. The dataset includes vaccination coverage data for children aged up to 36 months who had received specific vaccines at any time before the surveys according to their vaccination card, mothers’ verbal reports, and health facility records. We used vaccination coverage data of 1929 children aged 12–23 months from the 2016 EDHS in this study, resulting in a total number of 2004 children after taking into account stratification, cluster sampling, and differences in selection probability of participants through sample weights. For children who had visited a health facility but whose mothers were unable to present the vaccination cards, the health facility records were used to collect complementary vaccination data. This is an improved method of verifying the child vaccination records in the 2016 EDHS in comparison to the previous surveys and lowers the impact of recall bias.

### 2.2 Vaccination coverage

We primarily assessed full vaccination coverage among children aged 12–23 months, as defined by the current Ethiopian vaccination schedule, which builds upon the previous schedule of basic vaccination as defined by WHO guidelines and the DHS programme. Basic vaccination refers the receipt of one dose of Bacille Calmette-Guérin (BCG) vaccine, three doses of diphtheria-tetanus-pertussis-hepatitis B-Haemophilus influenzae type b (DTP3-HepB-Hib) pentavalent vaccine, three doses of polio vaccine, and one dose of measles vaccine among children aged 12–23 months [20]. Full vaccination refers to the receipt of all vaccines included in basic vaccination as well as three doses of pneumococcal conjugate vaccine (PCV3, introduced in 2011) and two doses of rotavirus vaccine (introduced in 2013) among children aged 12–23 months [14].

Vaccination coverage estimates were calculated using the approach outlined by the DHS programme [21]. In addition to assessing full vaccination coverage, we also assessed the coverage of single vaccinations and basic vaccination. Receipt of three doses of the combined diphtheria, tetanus toxoid, and pertussis vaccine (DTP3) is widely used as an indicator of immunisation programme performance and ability of families to repeatedly access immunisation services [13], [22].

### 2.3 Equity criteria

Based on the equity criteria according to the guidance on priority setting in health care (WHO GPS-Health), particularly those related to social groups, and the assessment of inequalities in childhood immunisation in ten Gavi priority countries by WHO, the following explanatory variables were chosen as relevant stratifiers for the measurement of inequalities in vaccination coverage: wealth index; maternal age at birth, maternal education and marital status; sex of household head; area and region of residence; ethnicity; religion; sex and birth order of the child [11], [23], [24]**.**

### 2.4 Equity impact analysis

The distribution of vaccination coverage by socioeconomic, geographic, maternal, and child characteristics was analysed to compute absolute and relative equity metrics, and the contribution of these characteristics to inequalities in vaccination coverage was estimated through logistic regression. Simple logistic regression was conducted to obtain unadjusted odds ratios for the association between each exposure variable and full vaccination coverage. The adjusted associations between full vaccination coverage as a binary outcome variable and selected exposure variables were assessed through multiple logistic regression (see Appendix A1). Wealth and maternal education related inequities in vaccination coverage were further explored through estimation of wealth and maternal-related concentration indices for vaccination coverage, and the concentration curve for full vaccination coverage by household wealth. Since vaccination status is a binary variable, the Wagstaff normalised concentration index was calculated. The concentration index is bounded between −1 and +1 with negative values indicating a pro-disadvantaged (pro-poor and progressive) distribution and positive values indicating a pro-advantaged (pro-rich and regressive) distribution of vaccination coverage in the population [26], [27], [28].

The analysis was conducted using Stata statistical software [25] by taking into account stratification, cluster sampling, and differences in selection probability of participants through sample weights (see Appendix A2).

### 2.5 Ethics approval

This study was approved by the ethics committee (Ref 16846) of the London School of Hygiene & Tropical Medicine. All authors had full access to all the data in the study and final responsibility for the decision to submit for publication.

## 3 Results

### 3.1 Vaccination coverage

After applying sample weights, 2004 children aged 12–23 months and their mothers were represented in the 2016 Ethiopia DHS. The mean DTP3 vaccination coverage among these children at national level was 53.2% [95% CI: 48.9–57.4]. Vaccination coverage for other single vaccines ranged from 49.1% [45.1–53.1] for PCV3 to 69.2% [65.5–72.8] for BCG vaccine. The coverage for basic vaccination (1-dose BCG, 3-dose DTP3-HepB-Hib, 3-dose polio, 1-dose measles vaccines) and full vaccination (basic vaccination plus 3-dose PCV3 and 2-dose rotavirus vaccines) was 38.5% [34.4–42.7] and 33.3% [29.4–37.2] respectively (see Table 1).

**Table 1:** **Vaccination coverage in Ethiopia at the national level and by rural and urban areas.** Vaccination coverage among children aged 12–23 months in Ethiopia and disaggregated by rural and urban areas of residence (mean coverage and 95% confidence intervals). Basic vaccination includes 1-dose BCG, 3-dose DTP3-HepB-Hib, 3-dose polio, and 1-dose measles vaccines. Full vaccination includes basic vaccination plus 3-dose PCV3 and 2-dose rotavirus vaccines.

**Vaccine**	**Coverage (%)**			
	**National** (N = 2004)	**Rural** (n_1_ = 1772)	**Urban** (n_2_ = 232)	**Urban-rural difference^1^**
BCG	69.2 (65.5, 72.8)	66.6 (62.6, 70.5)	88.8 (80.9, 96.7)	22.3 (13.5 31.1)
DTP-HepB-HiB	53.2 (48.9, 57.4)	49.7 (45.2, 54.2)	79.5 (68.2, 90.7)	29.8 (18.7, 41.8)
Polio	56.4 (52.4, 60.4)	53.4 (49.1, 57.7)	79.5 (69.9, 89.1)	26.1 (15.6, 36.6)
Measles	54.3 (50.2, 58.5)	51.5 (47.1, 55.9)	76.0 (63.6, 88.4)	24.5 (11.4, 37.5)
Basic vaccination	38.5 (34.4, 42.7)	35.1 (30.9, 39.4)	64.6 (51.8, 77.4)	29.5 (16.0, 43.0)
PCV	49.1 (45.1, 53.1)	46.0 (41.8, 50.2)	72.9 (61.2, 84.6)	26.9 (14.5, 39.3)
Rotavirus	56.0 (52.0, 59.9)	52.9 (48.8, 57.1)	79.1 (66.4, 91.8)	26.1 (12.8, 39.4)
Full vaccination	33.3 (29.4, 37.2)	29.7 (25.7, 33.6)	60.9 (48.2, 73.6)	31.2 (17.9, 44.5)

p < 0.001 for the respective differences in mean vaccination coverage between rural and urban areas

Among the 2004 children, 57.5% [53.0–62.0] had a vaccination card or health record and full vaccination coverage among these children was 56.2% [51.2–61.2]. Of the remaining 42.5% of children without a vaccination card or health record, full vaccination coverage was only 2.3% [0.6–4.0]. The urban–rural differences in vaccination coverage were significant with a pro-urban advantage (p < 0.001). The absolute differences in coverage were higher for DTP3, basic vaccination, and full vaccination at 29.8% [18.7–41.8], 29.5% [16.0–43.0], and 31.2% [17.9–44.5] respectively and lowest for BCG vaccination at 22.3% [13.5–31.1].

### 3.2 Equity impact: Vaccination coverage disaggregated by socioeconomic, geographic, maternal, and child characteristics

**Fig. 1:**
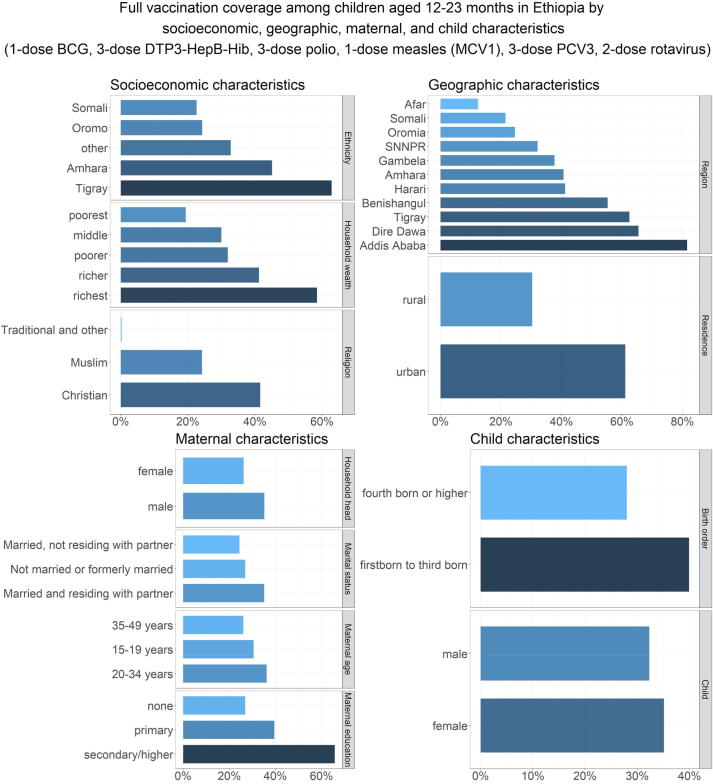
**Vaccination coverage in Ethiopia among children aged 12**–**23 months by socioeconomic, geographic, maternal,and child characteristics.** Full vaccination coverage (1-dose BCG, 3-dose DTP3-HepB-Hib, 3-dose polio, 1-dose measles (MCV1), 3-dose PCV3, and 2-dose rotavirus vaccines) in Ethiopia among children aged 12–23 months by socioeconomic (household wealth, religion, ethnicity), geographic (area of residence, region), maternal (maternal age at birth, maternal education, maternal marital status, sex of household head), and child (sex of child, birth order) characteristics.

**Fig. 2:**
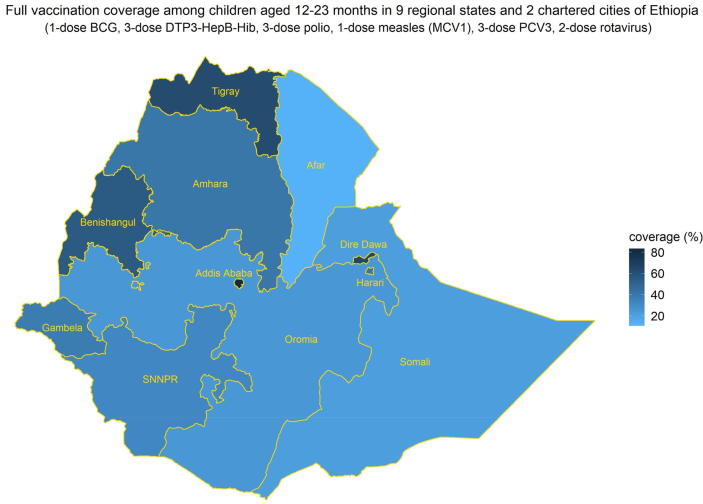
**Vaccination coverage in Ethiopia among children aged 12**–**23 months at the regional level.** Full vaccination coverage (1-dose BCG, 3-dose DTP3-HepB-Hib, 3-dose polio, 1-dose measles (MCV1), 3-dose PCV3, and 2-dose rotavirus vaccines) in Ethiopia among children aged 12–23 months in the nine regions and two chartered cities of Ethiopia.

The inequities in full vaccination coverage in Ethiopia disaggregated by socioeconomic, geographic, maternal, and child characteristics are illustrated in Table 2 and Fig. 1. With respect to household wealth status, full vaccination coverage was lowest among the poorest quintile (19.2% [13.0–25.5]) and highest in the richest quintile (58.3% [47.6–69.0]). Children in the richest quintile had 6 times the odds of full vaccination coverage compared with children in the poorest quintile (crude OR 5.87 [3.16–10.88]). With respect to religion, full vaccination coverage was relatively higher among Christian children at 40.8% [36.1–45.6] in comparison to Muslim children at 24.0% [18.1–30.0]. With respect to ethnicity, full vaccination coverage was relatively higher among Tigray children at 62.5% [53.8–71.2] and lowest among Somali children at 21.4% [11.8–31.0]. With respect to geographic characteristics, full vaccination coverage among children in urban and rural areas were 60.9% [48.2, 73.6] and 29.7% [25.7–33.6] respectively while it was highest in the capital Addis Ababa at 81.6% [73.7–89.5] and lowest in the Afar region at 12.4% [3.6–21.2] (see Fig. 2).

**Table 2:** **Inequities in full vaccination coverage in Ethiopia by socioeconomic, geographic, maternal, and child characteristics.** Inequities in full vaccination coverage (1-dose BCG, 3-dose DTP3-HepB-Hib, 3-dose polio, 1-dose measles (MCV1), 3-dose PCV3, and 2-dose rotavirus vaccines) among children aged 12–23 months by socioeconomic (household wealth, religion, ethnicity), geographic (area of residence, region), maternal (maternal age at birth, maternal education, maternal marital status, sex of household head), and child (sex of child, birth order) characteristics, based on simple logistic regression estimates of crude odds ratios (mean and 95% confidence intervals).

**Characteristics**	**Population in each subgroup n (N = 2,004)**	**Mean** **full coverage (%)**	**Odds Ratio**	**p-value**
**Household wealth** (quintiles)				
poorest (referent)	504	19.2 (13.0, 25.5)		<0.0001
poorer	396	31.0 (23.9, 38.1)	1.88 (1.13, 3.13)	
middle	450	29.9 (22.9, 36.9)	1.79 (1.04, 3.06)	
richer	366	39.7 (31.3, 48.1)	2.76 (1.62, 4.69)	
richest	288	58.3 (47.6, 69.0)	5.87values for 95% CI should be on the same level in the final version of the table (as in table 1) (3.16, 10.88)	
**Religion**				
Muslim (referent)	786	24.0 (18.1, 30.0)		<0.0001
Christian	1171	40.8 (36.1, 45.6)	2.18 (1.50, 3.18)	
Traditional and other	47	0.3 (-0.3, 0.9)	0.01 (0.00, 0.06)	
**Ethnicity**				
Somali (referent)	72	21.4 (11.8, 31.0)		<0.0001
Amhara	419	44.3 (36.0, 52.6)	2.92 (1.50, 5.68)	
Oromo	824	24.0 (17.9, 30.0)	1.16 (0.60, 2.24)	
Tigray	155	62.5 (53.8, 71.2)	6.11 (3.09, 12.10)	
other	534	32.2 (25.7, 38.7)	1.74 (0.91, 3.32)	
**Area of residence**				
rural (referent)	1772	29.7 (25.7, 33.6)		<0.0001
urban	232	60.9 (48.2, 73.6)	3.69 (2.10, 6.51)	
**Region**				
Afar (referent)	20	12.4 (3.6, 21.2)		<0.0001
Tigray	152	62.1 (52.8, 71.4)	11.60 (4.71, 28.56)	
Amhara	364	39.9 (30.8, 48.9)	4.69 (1.92, 11.47)	
Oromia	881	24.3 (17.9, 30.6)	2.27 (0.94, 5.48)	
Somali	76	19.9 (10.5, 29.3)	1.76 (0.65, 4.80)	
Benishangul	21	51.6 (40.1, 63.2)	7.56 (2.97, 19.24)	
Southern Nations, Nationalities & People	419	31.7 (24.1, 39.2)	3.28 (1.36, 7.93)	
Gambela	5	36.5 (25.8, 47.2)	4.07 (16.0, 10.35)	
Harari	5	40.4 (28.8, 52.0)	4.80 (1.87, 12.32)	
Addis Ababa	52	81.6 (73.7, 89.5)	31.39 (11.96, 82.41)	
Dire Dawa	9	65.5 (51.9, 79.0)	13.41 (4.89, 36.80)	
**Maternal age at birth** (years)				
15–19 (referent)	238	29.5 (20.8, 38.1)		0.03
20–34	1425	35.7 (31.5, 39.9)	1.33 (0.88, 2.02)	
35–49	341	25.9 (18.6, 33.2)	0.84 (0.49, 1.42)	
**Maternal education**				
none (referent)	1257	26.1 (22.0, 30.3)		<0.0001
primary	577	39.4 (33.2, 45.6)	1.84 (1.36, 2.49)	
secondary or higher	170	65.3 (51.3, 79.2)	5.31 (2.77, 10.17)	
**Maternal marital status**				
Not married or formerly married (referent)	107	26.8 (15.1, 38.5)		0.04
Married, not residing with partner	155	23.0 (14.1, 32.0)	0.82 (0.37, 1.81)	
Married and residing with partner	1742	34.6 (30.6, 38.6)	1.45 (0.80, 2.61)	
**Sex of household head**				
male (referent)	1705	34.7 (30.6, 38.8)		0.01
female	299	25.2 (18.5, 31.9)	0.64 (0.44, 0.91)	
**Sex of child**				
male (referent)	926	31.6 (26.4, 36.9)		0.36
female	1078	34.7 (29.8, 39.6)	1.15 (0.85, 1.55)	
**Birth order**				
4th born or higher (referent)	1028	27.5 (23.0, 32.0)		0.0001
First to 3rd born	976	39.4 (34.2, 44.5)	1.71 (1.31, 2.24)

With respect to maternal characteristics, full vaccination coverage was lowest among children whose mothers were between 15–19 or 35–49 years old at the time of birth (29.5% [20.8–38.1] and 25.9% [18.6–33.2] respectively), had no education (26.1% [22.0–30.3]), were not married or not residing with a partner (26.8% [15.1–38.5] and 23.0% [14.1–32.0] respectively), or who were living in female-headed households (25.2% [18.5–31.9]). Children whose mothers had secondary or higher education had 5 times the odds of full vaccination coverage compared to children whose mothers had no education (crude OR 5.31 [2.77–10.17]). With respect to child characteristics, full vaccination coverage was relatively highest among the first three born children at 39.4% [34.2–44.5], and there were no sex-related (male/female) discrepancies in full vaccination coverage.

#### 3.3 Wealth and maternal education related inequities in vaccination coverage

**Fig. 3:**
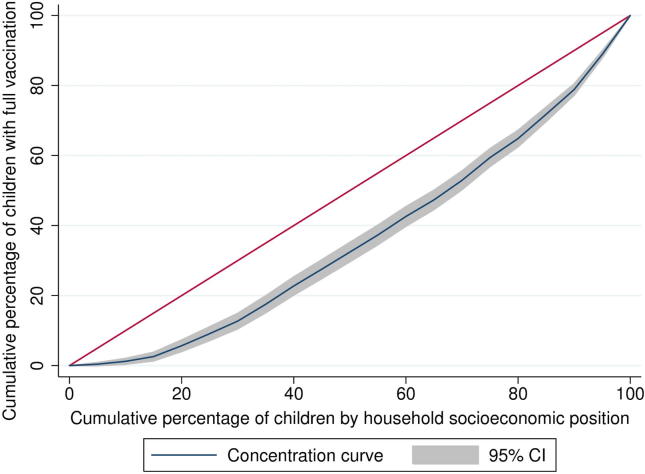
**Wealth related inequity in vaccination coverage in Ethiopia.** Concentration curve for full vaccination coverage (1-dose BCG, 3-dose DTP3-HepB-Hib, 3-dose polio, 1-dose measles (MCV1), 3-dose PCV3, and 2-dose rotavirus vaccines) in children aged 12–23 months by household wealth in Ethiopia.

Vaccination coverage in Ethiopia is characterised by a pro-rich distribution with respect to both household wealth and maternal education, with relatively higher vaccination coverage among children from richer households and higher maternal education (Table 3 and Fig. 3). The wealth and maternal education related concentration indices for basic vaccination were 0.28 and 0.24 respectively, and for full vaccination 0.30 and 0.23 respectively.

**Table 3:** **Wealth and maternal education related inequities in vaccination coverage for Ethiopia.** Wealth and maternal education-related concentration indices for vaccination coverage in children aged 12–23 months in Ethiopia. Basic vaccination includes 1-dose BCG, 3-dose DTP3-HepB-Hib, 3-dose polio, and 1-dose measles vaccines. Full vaccination includes basic vaccination plus 3-dose PCV3 and 2-dose rotavirus vaccines.

**Vaccine**	**Wealth-related** **concentration index**	**Maternal education-related** **concentration index**
BCG	0.25	0.17
DTP3-HepB-Hib	0.30	0.22
Polio	0.23	0.19
Measles	0.22	0.16
Basic vaccination	0.28	0.24
PCV3	0.24	0.18
Rotavirus	0.26	0.18
Full vaccination	0.30	0.23

The wealth-related concentration index for single vaccinations varied from 0.22 for measles vaccination to 0.30 for pentavalent vaccination, and the maternal education related concentration index varied from 0.16 for measles vaccination to 0.22 for pentavalent vaccination. Thus, with respect to both household wealth and maternal education, we infer a relatively higher equitable uptake of the single-dose measles vaccine (MCV1) in comparison to other childhood vaccines in Ethiopia.

#### 3.4 Equity impact: Socioeconomic, geographic, maternal, and child characteristics affecting vaccination coverage

**Fig. 4:**
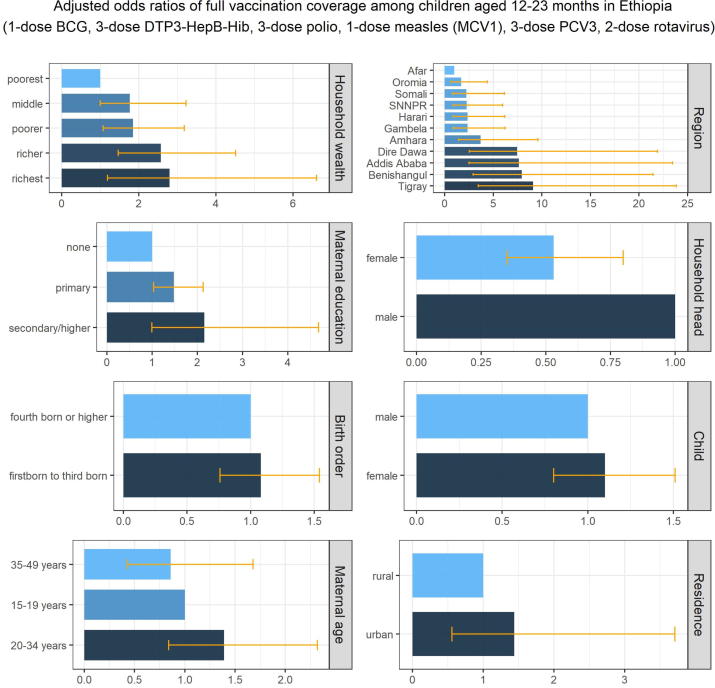
**Inequities in vaccination coverage in Ethiopia associated with socioeconomic, geographic, maternal, and child characteristics.** Inequities in full vaccination coverage (1-dose BCG, 3-dose DTP3-HepB-Hib, 3-dose polio, 1-dose measles (MCV1), 3-dose PCV3, and 2-dose rotavirus vaccines) among children aged 12–23 months associated with socioeconomic (household wealth), geographic (area of residence, region), maternal (maternal age at birth, maternal education, sex of household head), and child (sex of child, birth order) characteristics, based on multiple logistic regression estimates of adjusted odds ratios.

The inequities in full vaccination coverage in Ethiopia associated with socioeconomic, geographic, maternal, and child characteristics are illustrated in Table 4 and Fig. 4. Children in the richest wealth quintile households were 3 times more likely to have full vaccination coverage compared to children in the poorest wealth quintile households (adjusted OR 2.71 [1.15–6.41]). Children in Addis Ababa and Dire Dawa were 7 times more likely to have full vaccination coverage compared to children living in the Afar region (adjusted OR of 7.51 [2.45–23.01] and 7.43 [2.53–21.83] respectively). Children whose mothers had received primary education were 56% more likely to have full vaccination coverage compared to children whose mothers had no education (adjusted OR 1.56 [1.08–2.24]). Children living in female-headed households were 49% less likely to have full vaccination coverage compared to children living in male-headed households (adjusted OR 0.51 [0.33–0.77]).

**Table 4:** **Inequities in full vaccination coverage in Ethiopia associated with socioeconomic, geographic, maternal, and child characteristics.** Inequities in full vaccination coverage (1-dose BCG, 3-dose DTP3-HepB-Hib, 3-dose polio, 1-dose measles (MCV1), 3-dose PCV3, and 2-dose rotavirus vaccines) among children aged 12–23 months associated with socioeconomic (household wealth), geographic (area of residence, region), maternal (maternal age at birth, maternal education, sex of household head), and child (sex of child, birth order) characteristics, based on multiple logistic regression estimates of adjusted odds ratios (mean and 95% confidence intervals).

	**Adjusted Odds Ratios**	**p-value**
**Household wealth** (quintiles)		
poorest (referent)		
poorer	**1.77** (1.03, 3.04)	0.04
middle	1.74 (0.98, 3.10)	0.05
richer	**2.38** (1.34, 4.24)	0.003
richest	**2.71** (1.15, 6.41)	0.02
**Area of residence**		
rural (referent)		
urban	1.46 (0.57, 3.76)	0.44
**Region**		
Afar (referent)		
Tigray	**8.87** (3.38, 23.28)	<0.001
Amhara	**3.57** (1.37, 9.33)	0.009
Oromia	1.67 (0.65, 4.31)	0.28
Somali	2.02 (0.71, 5.76)	0.19
Benishangul	**6.81** (2.55, 18.17)	<0.001
Southern Nations, Nationalities & People	2.22 (0.84, 5.84)	0.11
Gambela	2.23 (0.85, 5.85)	0.10
Harari	2.28 (0.87, 5.99)	0.10
Addis Ababa	**7.51** (2.45, 23.01)	<0.001
Dire Dawa	**7.43** (2.53, 21.83)	<0.001
**Maternal age at birth** (years)		
15–19 (referent)		
20–34	1.46 (0.88, 2.43)	0.14
35–49	0.92 (0.47, 1.81)	0.82
**Maternal education**		
none (referent)		
primary	**1.56** (1.08, 2.24)	0.02
secondary/higher	**2.22** (1.03, 4.79)	0.04
**Sex of household head**		
male (referent)		
female	**0.51** (0.33, 0.77)	0.001
**Sex of child**		
male (referent)		
female	1.11 (0.81, 1.54)	0.51
**Birth order**		
4th born or higher (referent)		
First to 3rd born	1.08 (0.76, 1.53)	0.66

## 4 Discussion

Household wealth status, administrative regions, maternal education, and sex of household head were identified to be strongly associated with full vaccination coverage (1-dose BCG, 3-dose DTP3-HepB-Hib, 3-dose polio, 1-dose measles (MCV1), 3-dose pneumococcal (PCV3), and 2-dose rotavirus vaccines) after adjusting for background characteristics. Higher levels of full vaccination coverage were associated with children from richer households, urban regions of Addis Ababa and Dire Dawa, primary maternal education, and male-headed households in comparison to children from poorer households, rural regions of Afar and Somali, no maternal education, and female-headed households respectively.

The Ethiopian National Expanded Programme on Immunisation and the Global Vaccine Action Plan have set coverage goals of 90% at national level and 80% at district level by 2020 [5], [16]. However, full vaccination coverage in Ethiopia was well below the coverage goals at 33.3%, while the coverage of single vaccines ranged between 49.1% and 69.2% in 2016. Vaccination coverage in Ethiopia has a pro-advantaged regressive distribution with respect to both household wealth and maternal education, with relatively higher vaccination coverage among children from richer households and higher maternal education.

The findings of this study complement the findings of related studies. Tamirat et al inferred that female-headed households and rural dwellings were negatively associated with basic vaccination, while higher maternal education, employment, middle and rich economic status, ante-natal care follow up, and delivery at the health facility were positively associated with basic vaccination in Ethiopia [29]. Kinfe et al inferred that maternal education, husband employment, mother’s religion, maternal antenatal care visit, presence of vaccination document, region, and community antenatal care utilization were strongly associated with full vaccination [30]. Marefiaw et al found that age-inappropriate pentavalent 1–3 vaccinations were associated with being male sex, lack of telephone, lack of usual caretaker, unplanned pregnancy, missing pregnant women’s conference, decreasing birth order, and insufficient knowledge in Menz Lalo district of northeast Ethiopia [31]. Geremew et al detected seven significant space clusters with low MCV1 coverage with the primary, secondary, and tertiary clusters located in the Afar, Somali, and Gambella regions respectively [32]. Child age, first and third doses of pentavalent vaccination, secondary and above maternal education, and media exposure increased the odds of MCV1 vaccination while children with older maternal age had lower odds of MCV1 vaccination.

### 4.1 Ethiopia mini demographic and health survey 2019

The Ethiopia Mini Demographic and Health Survey 2019 was conducted between March and June of 2019 [33]. Data from vaccination card, mother’s report, and health facility records were collected for 1026 children aged 12–23 months for the following vaccinations: 1-dose BCG, 3-dose DTP3-HepB-Hib, 3-dose polio, 1-dose measles (MCV1), 3-dose PCV3 and 2-dose rotavirus. Additionally, data from 1028 children aged 24–35 months were collected for status on the second dose of measles vaccination (MCV2). While coverage estimates for basic vaccination are provided, full vaccination coverage is not included in the report as a summary measure. As primary data for the Ethiopia mini DHS 2019 is not yet available, based on the summary findings in the survey report, the coverage for all vaccines has improved slightly between 2016 and 2019. The inequities in single and basic vaccination coverage by wealth status of households, area of residence, region, and maternal education still persist in 2019.

### 4.2 Geographical factors

Nearly half of the contributing factors for under-immunisation in low and middle-income countries are immunisation systems related, with geographical factors affecting access and distance to health facilities being most common [34]. As further evidence of geographical barriers to accessing immunisation services, we identified significant differences in the odds of full vaccination coverage among the different regions of Ethiopia. Low population density and weak health infrastructure have been identified as challenges for routine immunisation services in Afar, Somali, and Gambella [14]. Afar and Somali regions in particular are dominated by pastoralist nomadic communities. Considering weak health infrastructure, distance to health facilities and mobility patterns of the population, routine immunisation services are difficult to deliver. Consequently, the current comprehensive National Immunisation Plan for 2016–2020 identifies pastoralist communities as populations at risk of not being reached by immunisation services. An example of an outreach approach are pre-arranged locations and dates for government health workers to meet with nomadic communities where vaccinations and basic health services are provided as well as scaling up the quantity of services [14], [16]. Improved understanding of spatiotemporal nomadic patterns will facilitate the service delivery of these outreach programmes to improve coverage and equitable uptake of vaccines among pastoralist communities. Also, taking into account the wide reach of immunisation programmes, improving equity in immunisation coverage through improved service delivery and outreach may simultaneously improve the delivery of other health interventions and preventive measures focused on child health [35].

### 4.3 Maternal education

Our results suggest that maternal education is associated with improved vaccination coverage in Ethiopia. Similar findings have been described for Ethiopia in multi-country assessments of inequities in vaccination coverage as well as in systematic review studies of 13 and 51 countries respectively [11], [34], [36], [37], [38], [39], [40]. Higher levels of maternal education raise awareness about the importance of vaccination and educated women choose health care services that generate better health [37], [41].

### 4.4 Household wealth status

Since vaccinations are provided free of charge at the public health facilities in Ethiopia, there are no financial barriers in the form of out-of-pocket payments for immunisation services. Also, political efforts have been made to reach rural and poor population groups through health extension workers based in communities [14], [16], [42]. Despite these efforts, the findings of this study still show an association of household wealth with improved vaccination coverage in Ethiopia.

Multi-country studies have found associations of children from households of lower socioeconomic status with lower vaccination coverage [34], [36], [39], [43]. In Ethiopia, there are likely to be additional financial barriers beyond out-of-pocket payments such as transportation costs and productivity loss from taking time off to access vaccination services and other non-financial barriers to vaccine acceptance among lower socioeconomic households, which warrants additional studies to explore and identify the underlying causal mechanisms.

### 4.5 Gender of household head

A systematic review on the influence of women’s empowerment on full immunisation coverage concluded that women’s agency measured as decision-making capacity is positively associated with their children’s vaccination coverage [44]. However, our study suggests that children living in female-headed households were less likely to have full vaccination coverage in comparison to children living in male-headed households in Ethiopia. These contrary findings warrant further exploration into associations between female agency and health-related decision-making. Sociocultural aspects relating to marital status and higher agency of married women living with their partner could be a potential explanation for the contrary findings.

### 4.6 Immunisation services

The differences in coverage between full vaccination and single vaccinations can be partly explained by factors relating to immunisation services and missed opportunities for vaccination. Within the three-tiered primary health care system, immunisation services are mainly delivered by health officers (nurses) who are based in health centres, health extension workers based at health posts, which are satellite posts of health centres, and the “health development army” – a workforce made up of volunteers who promote immunisations in their villages and help organise outreach activities in their communities. Low monitoring and supervision capacities prevent a focus on immunisation service delivery and result in insufficient feedback given to health staff. Despite a government policy requiring health facilities to offer immunisation services on a daily basis, a 2016 survey found that only 51% of health centres and 11% of health posts offered daily immunisation services in Ethiopia due to shortage of qualified health workers and challenges relating to vaccine supply and cold chain management [45]. Even though 31% of health facilities had refrigerators, only 11% had adequate refrigerator temperature. The cold chain rehabilitation and expansion plan aims to address these challenges by improving both logistic and storage aspects of vaccine distribution [14].

### 4.7 Missed opportunities for vaccination

Missed opportunities for vaccination occur when children or mothers receive health services, but the children miss out on vaccination [46], [47]. Missed opportunities to administer all vaccines scheduled at the same visit can indicate difficulties in vaccine supply, health workers’ reluctance to administer multiple vaccines simultaneously, and knowledge gaps in identifying which vaccines are due [48]. More than 50% of mothers and children in Ethiopia received one or more health interventions of antenatal care, postnatal care, vitamin A supplement, skilled birth attendants, and insecticide-treated bed-nets but have failed to have their children fully vaccinated [49]. Also, supplementary immunisation activities for polio, measles, tetanus, and meningococcal A vaccinations during 2011–2014 [16], which are mass-immunisation campaigns targeted at children otherwise missed by routine services, could lead to a one-sided focus on specific vaccines while missing out on other routine vaccinations [50]. On the positive front, there was a 31 percentage point increase in basic vaccination coverage among children of mothers who received postnatal care in Ethiopia in comparison to the children of mothers who had not received postnatal care [49], which provides evidence that mothers receiving health services are more likely to vaccinate their children.

### 4.8 Gavi support for Ethiopia

A goal of Gavi’s 2021–2025 strategy is “to bring a much stronger focus on reaching those most marginalised, by strengthening primary health care systems, building and sustaining community demand, and using innovation to ensure that immunisation services reach these children”. This is in line with the ‘leave no one behind’ and ‘reaching the furthest behind first’ principles of the SDGs [8], [51]. In a situation where Ethiopia is facing a phase-out of donor support for several programmes which are aimed at improving vaccination coverage in underserved administrative regions and zones, such as the Routine Immunisation Improvement Plan [14], [16], such a focus and subsequent support by Gavi could be crucial in improving vaccination coverage among the children who are currently not reached by immunisation services.

Our study findings add to the evidence base for performance monitoring and measurement against vaccination targets, and the analytical insight to inform Gavi strategies to increase immunisation access in Ethiopia [11], [19], [29], [30], [31], [32]. Specifically, targeted programmes to increase vaccination among children of poorer households, rural regions of Afar and Somali, no maternal education, and female-headed households will improve vaccination coverage and equity outcomes in Ethiopia. While the lack of vaccination cards and health records signal a bottleneck for immunisation access, they assist in identifying the subgroups of under-immunised children. Targeted programmes to reach these under-immunised children through mass-immunisation campaigns and outreach activities in rural areas will further improve vaccination coverage and equity outcomes.

### 4.9 Limitations and future directions

The vaccination coverage data collected in the Ethiopia DHS 2016 were subject to several sources of bias. Mothers’ verbal reports for vaccination status of their children were subject to recall bias. As these data were collected as part of the women’s questionnaire, only the children whose biological mothers were alive at the time of survey were included, and thereby subject to selection bias.

While we did not analyse missed opportunities for vaccination among different subgroups in Ethiopia, such a disaggregated analysis will be valuable for development and implementation of targeted strategies to improve vaccination coverage among the missed out children who receive other health services. Also, geospatial analysis of vaccination coverage will be beneficial for subnational targeting and reaching zero-dose and under-immunised children to improve coverage and equity outcomes. There are differences in vaccination coverage estimates between DHS, WUENIC, and official estimates of national authorities that should be taken into account in guiding policy and practice to improve vaccination coverage and equity in Ethiopia (see Appendix A3).

Qualitative studies are further needed to understand the specific barriers facing the parents of under-immunised children to access immunisation services. This will facilitate the design and implementation of targeted programmes to overcome the identified barriers and improve vaccination coverage among children in underserved communities.

### 4.10 Conclusion

In the comprehensive multi-year plan 2016–2020 of the Ethiopia National Expanded Programme on Immunisation, the Ministry of Health identifies inequities in access to immunisation services, as well as regional disparities, as major weaknesses in immunisation service delivery. The Ministry of Health has proposed to tackle these inequities by implementing all components of the WHO/UNICEF “Reaching every community” approach during 2016–2020 [16]. Our study contributes to this approach by analysing childhood vaccination coverage in Ethiopia by socioeconomic, geographic, maternal, and child characteristics to identify population subgroups with low vaccination coverage – children from poorer households, rural regions of Afar and Somali, no maternal education, and female-headed households. This evidence will facilitate the design and development of targeted approaches to reach the under-immunised children of different socioeconomic, geographic, maternal, and child characteristics to improve vaccination coverage and equity outcomes in Ethiopia.

### CRediT authorship contribution statement

**Anne Geweniger:** Conceptualization, Methodology, Software, Validation, Formal analysis, Investigation, Data curation, Writing - original draft, Writing - review & editing, Visualization. **Kaja M. Abbas:** Conceptualization, Methodology, Resources, Writing - review & editing, Supervision, Project administration, Funding acquisition.

## Declaration of Competing Interest

The authors declare that they have no known competing financial interests or personal relationships that could have appeared to influence the work reported in this paper.

## Figures and Tables

**Table A3:** 1. DHS, WUENIC, and official estimates of vaccination coverage in Ethiopia.

**Vaccine and dose**	**2016 DHS (children aged 12**–**23 months)**	**WUENIC 2014 estimates** **(unadjusted)**	**WUENIC 2014 estimates (adjusted)**	**Official country estimates (2014)**
BCG	69	75	69	78
DTP1	73	86	73	89
DTP3	53	77	61	84
Polio	56	75	68	84
Measles (MCV1)	54	70	54	81
PCV3	49	76	55	82
Rotavirus	56	63	56	70

## Appendix

### Appendix A 1. Model development and variable selection for multiple logistic regression

Simple logistic regression was conducted to assess associations of full vaccination coverage with socioeconomic (household wealth, religion, ethnicity), geographic (area of residence, region), maternal (maternal age at birth, maternal education, maternal marital status, sex of household head), and child (sex of child, birth order) characteristics.

Multiple logistic regression was then conducted to assess adjusted associations between full vaccination coverage and socioeconomic (household wealth), geographic (area of residence, region), maternal (maternal age at birth, maternal education, sex of household head), and child (sex of child, birth order) characteristics. Maternal marital status was excluded in the multiple logistic regression analysis due to collinearity with sex of household head – in households where married women are living with a partner, household head was likely to be male. Religion and ethnicity were excluded from the multiple logistic regression analysis due to collinearity with region – administrative regions in Ethiopia are inhabited by major ethnic groups specific to that region, and the predominant religions are specific to the ethnic groups.

Joint hypothesis testing using a Wald test was performed for categorical exposure variables with two or more subgroups to test the null hypothesis of no overall association between the variable and full vaccination coverage. Effect modification between selected exposure variables and full vaccination coverage was investigated by including interaction terms in the regression model. Interaction between the following variables was tested.

• household economic status (i.e. wealth quintile) and area of residence

• household economic status and maternal age

• household economic status and maternal education

• maternal age and maternal education

Due to small sample size in some subgroups, the exposure groups of maternal education were combined to create a binary variable and thus increase the power of testing for interaction.

## Appendix A 2. Reproducible analysis

The 2016 Ethiopia DHS data file (ETKR71DT.ZIP) used in this study is accessible on the DHS website (https://dhsprogram.com/data/dataset/Ethiopia_Standard-DHS_2016.cfm) and available to registered users. The Stata codes used for the study analysis are available upon request from the corresponding author (anne.geweniger1@alumni.lshtm.ac.uk).

## Appendix A 3. Vaccination coverage estimates for Ethiopia

There are differences in vaccination coverage estimates for 2016 between Ethiopia DHS, WUENIC (WHO/UNICEF Estimates of National Immunization Coverage) and official estimates of national authorities, as shown in Table A3. DHS is based on population-based household surveys among children aged 12–23 months, and collects information on vaccination history from documented evidence or caregiver recall. WUENIC uses both administrative as well as survey data which are assessed for potential biases, and the final estimates are only derived after consultation with local partners in the respective countries [52], [53]. Official estimates reported by national authorities reflect their assessment based on administrative coverage, survey-based estimates, other data sources or adjustments. The differences in vaccination coverage estimates between different sources should be taken into account in guiding policy and practice to improve vaccination coverage and equity in Ethiopia.
